# Simultaneous gastroepiploic artery to right coronary artery bypass and trans-catheter aortic valve implantation: case series

**DOI:** 10.1186/s44215-024-00185-z

**Published:** 2025-01-06

**Authors:** Kentaro Honda, Teruaki Wada, Hideki Kunimoto, Yoshiharu Nishimura

**Affiliations:** 1 Department of Thoracic and Cardiovascular Surgery, Wakayama Medical University, 811-1 Kimiidera, Wakayama City, Wakayama, 641-8509 Japan; 2 Department of Cardiovascular Medicine, Wakayama Medical University, Wakayama, Japan

**Keywords:** Minimally invasive CABG, GEA-PDA bypass, TAVI

## Abstract

Patients with coronary artery disease undergoing trans-catheter aortic valve implantation (TAVI) often receive TAVI alone. However, in cases of severe coronary lesions or anticipated difficulty in coronary access post-TAVI, percutaneous coronary intervention or coronary artery bypass grafting may be necessary. We performed simultaneous gastroepiploic artery to posterior descending artery bypass and TAVI in two patients with severe calcification of the right coronary artery ostium which is unsuitable for percutaneous intervention. The procedure was conducted through an upper median laparotomy, avoiding a full sternotomy. Patients were discharged on postoperative days 6 and 9, respectively. By combining minimally invasive techniques and avoiding median sternotomy, we achieved favorable outcomes without compromising the less invasive nature of TAVI. This approach demonstrates the potential for tailored, hybrid procedures in high-risk patients with concomitant aortic valve disease and complex coronary lesions.

**Supplementary Information:**

The online version contains supplementary material available at 10.1186/s44215-024-00185-z.

## Background

We report two cases of simultaneous gastroepiploic artery to right coronary artery (RCA) bypass and trans-catheter aortic valve implantation (TAVI) in elderly patients. TAVI is a commonly used treatment which has become simplified over time. Even in cases of coronary artery disease, isolated TAVI is often performed without coronary intervention [1]. Clinical outcomes reportedly did not significantly differ in a previous study among patients with concomitant significant coronary artery disease who underwent TAVI according to whether or not they underwent preceding percutaneous coronary intervention (PCI) [2]. However, some coronary artery lesions are reportedly difficult to treat after TAVI [3]. In such cases, coronary intervention may be considered at the time of TAVI, taking into account the possibility of future coronary intervention. Our two patients had highly calcified stenotic lesions of the RCA orifice in which PCI was considered unsuitable. We therefore performed simultaneous TAVI and right gastroepiploic artery to posterior descending artery (rGEA and PDA) bypass through an upper midline laparotomy approach with off-pump technique.

## Case presentation

**Fig. 1:**
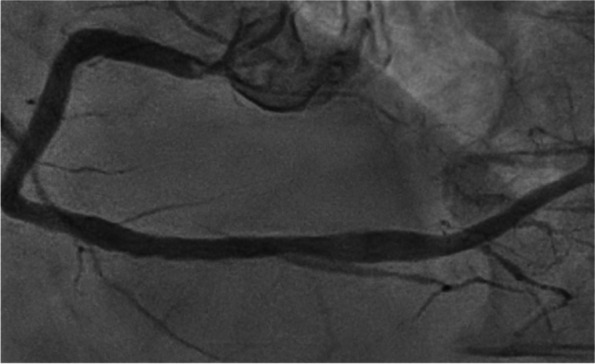
Severe calcified lesion of the RCA orifice

Case 1 was an 87-year-old male patient (height, 155.4 cm; weight, 55.8 kg; body surface area, 1.51 m^2^) with severe aortic valve stenosis. Echocardiography revealed reduced aortic valve area (0.59 cm^2^). Peak and mean pressure gradients were 63 and 42 mmHg, respectively. Preoperative coronary angiography revealed a severe, calcified lesion of the RCA orifice (Fig. 1) that was unsuitable for PCI. We planned rGEA-PDA bypass and trans-femoral TAVI.

**Fig. 2:**
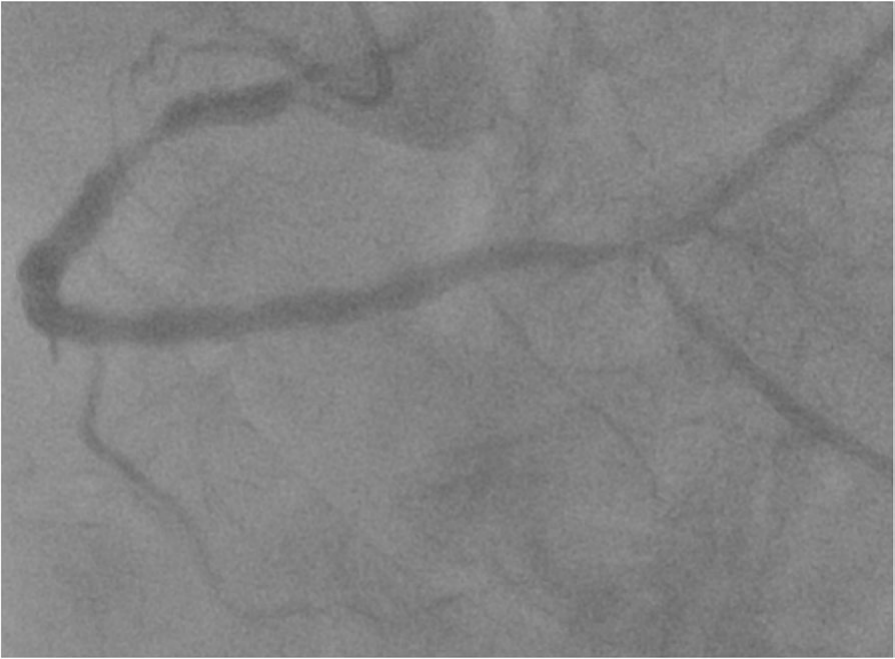
Severe calcified lesion of the proximal RCA (#1 and #2)

Case 2 was a 78-year-old male patient (height, 161.4 cm; weight, 74.2 kg; body surface area, 1.82 m^2^) with dyspnea and chest discomfort during mild exertion. Echocardiography revealed reduced aortic valve area (0.71 cm^2^); peak and mean pressure gradients were 63 and 42 mmHg, respectively. Computed tomography revealed an extensive calcified and stenotic lesion of bilateral iliac arteries and aortic aneurysm of the arch and calcified ascending aorta. Coronary angiography revealed severe calcified lesion of the proximal RCA (#1 and #2) (Fig. 2). We planned simultaneous off-pump rGEA-PDA bypass and trans left subclavian artery approach TAVI.

### rGEA harvest

Surgery in both cases was performed under general anesthesia with the patients in the supine position. A soft cushion was placed in the back so that the upper abdomen would be warped. Upper median laparotomy with a 9-cm skin incision was performed, and the xiphoid process was removed. The peritoneal cavity was opened, and the stomach was towed out of the body with gauze. The rGEA was inspected and palpated before harvesting. The rGEA was secured in the vessel loop, and using an ultrasonic scalpel (Harmonic Scalpel; Ethicon Endo-Surgery, Cincinnati, OH), the rGEA was harvested in a semi-skeletonized fashion along with the greater curvature of the stomach, proximally to the gastroduodenal artery and distally to the very end of the rGEA, following a previously-reported procedure [4].

### rGEA- PDA bypass

We dorsally incised the diaphragm to identify the PDA. The liver was carefully handled to avoid damage during this process. We sutured the diaphragm in 6–8 places with a surgical towel, and the towel was pulled caudally to rotate the heart position.

**Fig. 3:**
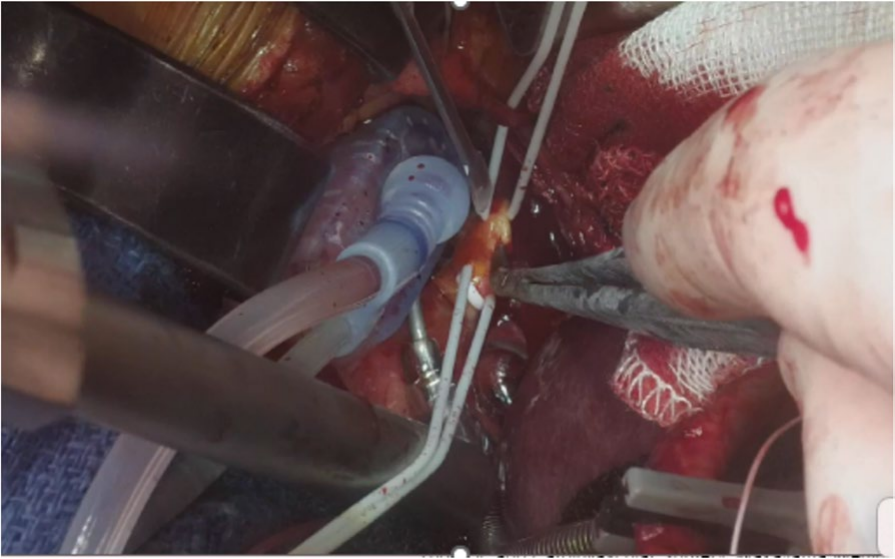
Expansion of visual field with Octopus NUVO and tentacles NEO

A Kent retractor was also placed over the left hypochondria and pulled upward to create space in the operative field. We used a multi-suction heart positioner to manage the position of the heart (Tentacles Neo Heart Positioner, SB-Kawasumi Laboratories, Inc. Kanagawa, Japan). One or two arms of the heart positioner were placed on the surface of the inferior wall and it was pulled upwards to enable close observation of the PDA. Octopus Nuvo (Medtronic, Inc. Minneapolis, USA) was also used to stabilize the anastomosis site, and in situ rGEA was anastomosed in a parallel fashion. In Case 2, it was difficult to achieve the optimal anastomotic field with an upper median laparotomy only. The skin incision was therefore extended, and we performed lower partial hemi-sternotomy to enable a good operative view (Fig. 3).

TAVI was followed in both cases. In Case 1, we performed trans-femoral TAVI with 23 mm SAPIEN 3 ultra resilia (Edwards Lifesciences, Irvine, CA, USA). In Case 2, we performed trans-subclavian TAVI with 26 mm SAPIEN 3 ultra resilia. Total operation times were 200 and 360 min, respectively. Patients re-started walking on postoperative day 2 in both cases and were discharged on postoperative days 6 and 9, respectively, without any problems (Video 1).

## Discussion

TAVI is increasingly becoming minimally invasive, local anesthesia is gradually replacing general anesthesia, and comparatively simpler TAVI procedures have become the norm [2]. TAVI can also be safely performed without coronary intervention, even in patients with complicated coronary artery lesions. On the other hand, the approach to the right coronary artery after TAVI valve implantation is often difficult.

Reasons for the difficult coronary access after TAVI include valve frame obstruction, valve type (self-expandable valves tend to be more difficult to access than balloon expandable valves), high implantation, anatomical reason (narrow sinus of Valsalva and low coronary take-off), interaction with prosthetic valve leaflets, and altered aortic root pathology after TAVI [3]. In patients with small stature, such as Japanese and other Asian populations, it can sometimes be challenging to access the RCA after TAVI. In both cases described in this paper, due to severe calcification and the lesions being located at the orifice, it was anticipated that PCI following TAVI would be difficult. We therefore opted to perform simultaneous minimally-invasive CABG in these cases.

There have been reports on the utility of the rGEA [5], on improved harvesting techniques [6], and around the year 2000, there were reports on the minimally-invasive GEA-PD bypass via upper median laparotomy [7, 8]. However, in recent years, there have been comparatively few reports on such methods. We have found the addition of lower partial sternotomy and the use of a Kent retractor to be beneficial when the visual field is poor.

## Conclusion

Minimally invasive surgery is suggested to be beneficial in elderly, frail patients who are candidates for TAVI. Wider investigation is required, but our surgical method appears to be useful for minimally invasive revascularization of the RCA.

## Supplementary Information

Additional file 1

## Data Availability

Data sharing is not applicable.

## Abbreviations

PCI: Percutaneous coronary intervention
PDA: Posterior descending artery
RCA: Right coronary artery
rGEA: Right gastroepiploic artery
TAVI: Trans-catheter aortic valve implantation
